# *Chrysanthemum zawadskii* var. *latilobum* Flower Essential Oil Reduces MRSA Pathogenicity by Inhibiting Virulence Gene Expression

**DOI:** 10.3390/molecules30030553

**Published:** 2025-01-25

**Authors:** Ji-Hee Kim, Bog-Im Park, Young-Hoi Kim, Ji-Su Yoon, Na-Young Choi, Kang-Ju Kim

**Affiliations:** 1 Department of Convergence Technology for Food Industry, School of Food, Wonkwang University, Iksan 54538, Jeonbuk, Republic of Korea; ckhae@naver.com; 2 Transdisciplinary Major in Learning Health Systems, Department of Health and Safety Convergence Science, Graduate School, Korea University, Seoul 02841, Republic of Korea; cholong0326@naver.com; 3 Department of Food and Nutrition, School of Food, Kunsan National University, Kunsan 54150, Jeonbuk, Republic of Korea; parkbogim@hanmail.net; 4 Department of Food Science and Technology, College of Agriculture and Life Sciences, Jeonbuk National University, Jeonju 54896, Jeonbuk, Republic of Korea; yhoi1307@hanmail.net; 5 Clothing and Textiles Major, College of Education, Wonkwang University, Iksan 54538, Jeonbuk, Republic of Korea; 6 Department of Oral Microbiology, School of Dentistry, Wonkwang University, Iksan 54538, Jeonbuk, Republic of Korea

**Keywords:** *Chrysanthemum zawadskii*, MRSA, antimicrobial, biofilm, virulence

## Abstract

The essential oil extracted from the flowers of *Chrysanthemum zawadskii* var. *latilobum* (Maxim.) Kitam (CZEO), family Asteraceae, was investigated to determine its ability to inhibit the pathogenicity of methicillin-resistant *Staphylococcus aureus* (MRSA). The chemical composition of CZEO was analyzed using gas chromatography–flame ionization detector and gas chromatography–mass spectrometry, and 88 compounds were identified and categorized as monoterpenes (68.82%), sesquiterpenes (17.82%), and others (5.01%). CZEO inhibited MRSA floating cell growth, acid production, and biofilm formation in a concentration-dependent manner. Furthermore, confocal laser scanning and scanning electron microscopy confirmed that the CZEO treatment decreased MRSA viability and notably reduced the three-dimensional density of the biofilm. Real-time PCR demonstrated that the mRNA expression of the MRSA gene A (*mecA*), accessory gene regulator A (*agrA*), staphylococcal accessory regulator A (*sarA*), and staphylococcal enterotoxin A (*sea*), which are pivotal genes implicated in MRSA pathogenicity, declined in a concentration-dependent manner following the CZEO treatment compared with the control. Thus, CZEO appeared to directly target the pathogenicity MRSA regulators. These findings substantiate the potential of CZEO as a natural antimicrobial agent for preventing MRSA infections.

## 1. Introduction

Methicillin-resistant *Staphylococcus aureus* (MRSA) is a major global public health challenge that causes various illnesses, including food poisoning, pneumonia, and skin and soft tissue infections. In recent years, the prevalence of MRSA infections in both hospital and community settings has alarmingly increased [1]. Considering the seriousness of this situation, the 79th United Nations General Assembly committed to reducing antibiotic-resistant mortality by 10% by 2030. Furthermore, the Food and Agriculture Organization of the United Nations (FAO), World Health Organization (WHO), United Nations Environment Programme (UNEP), and World Animal Health Organization (WOAH) have highlighted the need for enhanced resistance monitoring through a Global Antimicrobial Resistance Surveillance System [2].

The continuous administration of methicillin results in the acquisition of multidrug resistance in MRSA, which limits treatment options. Antibiotic resistance is caused by the transfer of plasmids, mobile genetic elements, and mutations in chromosomal genes through the exchange of genetic material between bacteria [3]. MRSA is resistant to beta-lactam drugs because the *mecA* gene in the staphylococcal cassette chromosome *mec* produces the penicillin-binding protein-2α (PBP2α) [4].

According to the guidelines of the Infectious Diseases Society of America, vancomycin or daptomycin is the primary treatment for severe infections, serious infections, endocarditis, and sepsis. Second-line therapies for skin and soft tissue infections include linezolid and clindamycin. Linezolid has also been recommended for the treatment of MRSA pneumonia. Second-line agents are often orally administered and may be selected when vancomycin resistance is a concern [5]. The declining efficacy of existing antibiotics has driven the development of new antimicrobials. Vancomycin has been used to treat gram-positive bacteria, including MRSA, since it was authorized by the U.S. Food and Drug Administration (FDA) in 1958. However, vancomycin-resistant *S. aureus* isolates have emerged, limiting the effectiveness of the treatment [3,6]. Vancomycin binds to D-Ala-D-Ala in the cell wall, thereby inhibiting cell wall synthesis. However, the *vanA* gene modifies vancomycin to D-Ala-D-Lac, resulting in resistance [3,7]. In 2017, the European Medicines Agency’s Committee for Medicinal Products for Human Use (CHMP) identified several adverse effects of vancomycin, including acute hypersensitivity reactions, ototoxicity, nephrotoxicity, peritonitis, and cardiac depression. The CHMP emphasizes the importance of dose adjustments and regular monitoring when prescribing vancomycin [8]. In 2003, the FDA approved daptomycin for the treatment of soft tissue infections, and in 2006, it was approved for the treatment of MRSA. Daptomycin functions as a sterilizing agent by depolarizing cell membranes; however, *mprF* mutations alter membrane charge, thereby conferring resistance. Adverse effects include myotoxicity and interactions with pulmonary surface-active agents, rendering this drug unsuitable for treating pneumonia. Linezolid was approved by the FDA in 2000 for the treatment of infections caused by MRSA and vancomycin-resistant *enterococci*. Linezolid binds to the 50S ribosome and inhibits protein synthesis; however, resistance is induced by *23S* ribosomal RNA (rRNA) mutations or *cfr* mutations. The primary adverse effect is thrombocytopenia, and an increased risk of serotonin syndrome is observed when linezolid is combined with monoamine oxidase inhibitors [3,7]. Clindamycin is a lincosamide antibiotic that binds to 50S ribosomes and inhibits protein synthesis [9]. However, clindamycin-resistant MRSA strains are emerging [10,11]. The development of clindamycin resistance is attributed to the methylation of *23S* rRNA, which impedes the binding of the antibiotic to the ribosome, or the *linA* gene product’s modification of the antibiotic, leading to its inhibition [9]. Adverse effects include diarrhea and pseudomembranous colitis [12]. In rare instances, hepatotoxicity has been documented, manifesting as transient elevations in serum aminotransferase levels and acute specific liver injury within 1–3 weeks following the commencement of therapy [13]. Anaphylaxis, which is an acute hypersensitivity reaction, may also occur [14].

Considering the limitations of conventional antibiotics, the development of novel alternative antimicrobials, such as natural essential oils, is gaining attention. *Chrysanthemum zawadski* var. *latilobum* (Maxim.) Kitam (*C. zawadski* var. *latilobum*) is an autumn-flowering species of the Asteraceae family endemic to Northeast Asia. The flowers of this plant have been used as traditional tea in Korea [15]. In traditional Korean medicine, this plant is used to treat respiratory diseases (pneumonia and bronchitis), coughs, colds, and gastrointestinal disorders. Additionally, the ancient Chinese medicine Boncho Kangmok is listed as a herbal remedy for headaches, dermatitis, and indigestion [16,17]. *C. zawadski* var. *latilobum* exhibits a range of bioactivities, including anti-inflammatory, antioxidant, and anti-allergic properties [15,17,18,19]. These findings suggest the presence of bioactive substances in the form of essential oil in this species.

The objective of this study was to analyze the chemical composition of *C. zawadski* var. *latilobum* flower essential oil (CZEO) using gas chromatography–flame ionization detection (GC–FID) and gas chromatography–mass spectrometry (GC–MS) and ascertain its antibacterial activity against MRSA and inhibitory effect on biofilm formation. The effects of CZEO on the expression of antibiotic resistance-related (*mecA*), virulence regulatory (*agrA* and *sarA*), and enterotoxin (*sea*) genes in MRSA were investigated at the molecular level.

## 2. Results

### 2.1. Analysis of the Chemical Composition of CZEO

GC–FID and GC–MS analyses revealed that *CZEO* contained 88 compounds, representing 91.65% of the total composition (Table 1). Camphor, the oxidized form of the monoterpene with a ketone group attached to the C_10_H_16_ backbone, had the highest concentration at 39.69%. The next most prevalent monoterpene was camphene (6.33%), followed by notable quantities of bornyl acetate (5.42%), *cis*-chrysanthenol (2.79%), 1,8-cineole (2.66%), borneol (2.23%), and α-pinene (1.15%), which are all characterized by a C_10_H_16_ backbone with two isoprene units. Among the sesquiterpenes, α-cadinol, which contains an alcohol group, was the most prevalent at 5.77%. Other sesquiterpene compounds were identified, including torreyol (1.44%), spathulenol (1.3%), and 1,5-epoxysalvial-4(14)-ene (1.22%), which contain three isoprene units and C_15_H_24_ as the basic skeleton. The overall chemical composition was dominated by monoterpenes (68.82%), followed by sesquiterpenes (17.82%) (Table 2). In the trace components, alkanes with carbon chain lengths ranging from C_12_ (dodecane) to C_25_ (pentacosane) were identified, accounting for 0.04–0.66% of the total composition.

### 2.2. Antimicrobial Activity of CZEO Against Planktonic MRSA

**Figure 1:**
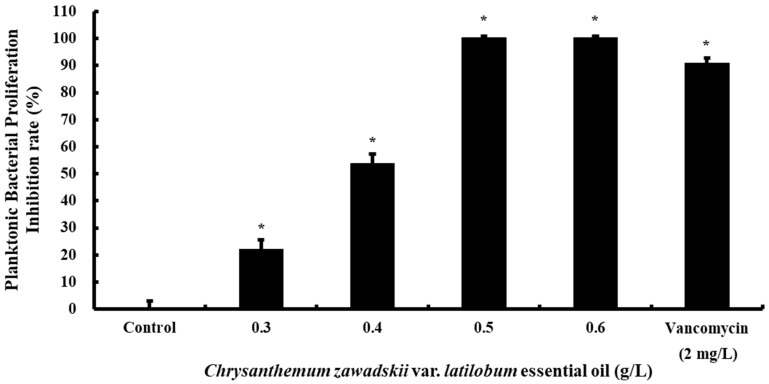
*Chrysanthemum zawadskii* var. *latilobum* essential oil (CZEO) deters methicillin-resistant *Staphylococcus aureus* (MRSA) proliferation. The MRSA strain ATCC 33,591 was seeded into brain heart infusion (BHI) broth at a concentration of 5 × 10^5^ CFU/mL and co-cultured with CZEO at a concentration of 0.3–0.6 g/L at 37 °C for 24 h. The turbidity of the cultured beta-lactam antibiotic-resistant strains was assessed by measuring at a wavelength of 550 nm. Results are expressed as means ± standard deviations from independent measurements. Vancomycin at a concentration of 2 mg/L was separately added to BHI broth as a positive control, and measurements were carried out using the same methods as the experimental group. * *p* < 0.05, statistically significant at 95% confidence level.

The effect of CZEO on planktonic MRSA was investigated by monitoring changes in bacterial density and acidity. To assess the influence of CZEO on microbial proliferation and acid secretion, various concentrations of CZEO, ranging from 0.3 to 0.6 g/L, were tested under control conditions. These findings demonstrate that CZEO markedly inhibited the proliferation of MRSA in a concentration-dependent manner. CZEO treatments at concentrations of 0.3 and 0.4 g/L demonstrated growth inhibition rates of approximately 22.13% and 53.83%, respectively, compared with that of the control (Figure 1). At higher concentrations of 0.5 and 0.6 g/L, bacterial growth was almost completely inhibited, indicating that CZEO has a bacteriostatic effect. Vancomycin, as the positive control, exhibited an inhibition rate of approximately 90.96%, suggesting that CZEO possesses antibacterial activity with the potential to substitute for vancomycin.

This study aimed to assess the potential of CZEO to deter MRSA proliferation, with a particular focus on its capacity to modulate acid production by MRSA. Table 3 illustrates the effect of CZEO on the production of organic acids by MRSA strain ATCC 33591. The fluctuations in pH of bacterial cultures exposed to varying concentrations (0.3, 0.4, 0.5, and 0.6 g/L) of CZEO and negative and positive controls were evaluated. The initial pH of all groups before incubation was not significantly different. Following a 24 h incubation period, the pH of the negative control group decreased to 5.96 ± 0.01, indicating acid production by MRSA. In contrast, the decrease in pH was mitigated by the CZEO-treated group in a concentration-dependent manner. After incubation, the measured pH was 6.53 ± 0.02 (inhibition rate of 38.58%) in the 0.3 g/L treatment, 6.80 ± 0.04 (inhibition rate of 56.54%) in the 0.4 g/L treatment, 7.13 ± 0.03 (inhibition rate of 79.38%) in the 0.5 g/L treatment, and 7.40 ± 0.01 (inhibition rate of 96.90%) in the 0.6 g/L treatment. Of note, the pH of the 0.6 g/L treatment approached the initial pH. The pH of the vancomycin (2 mg/L) treatment, which represented the positive control, was 7.41 ± 0.02 (Inhibition rate of 96.45%), indicating that high concentrations of CZEO inhibited MRSA acid-secreting activity.

### 2.3. Inhibitory Effect of CZEO on MRSA Biofilms: Quantitative Analysis and Observation of Microstructural Changes

**Figure 2:**
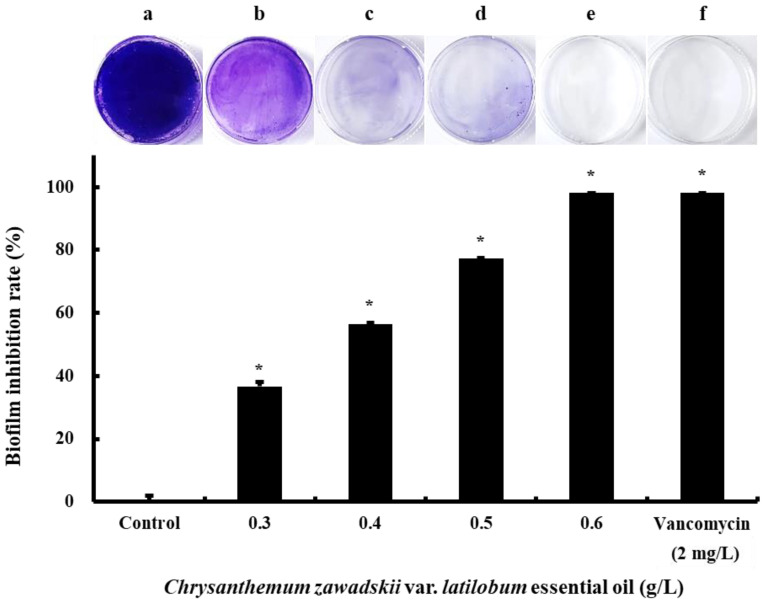
CZEO suppresses the formation of MRSA biofilm. Experiments were conducted to investigate the formation of MRSA biofilms utilizing 35 mm cell culture plates and crystal violet staining. BHI medium containing 1% glucose was utilized, and MRSA was inoculated after CZEO pre-treatment and incubated at 37 °C for 24 h. The formed MRSA biofilms were stained with a 1% crystal violet solution. After staining, 30% acetic acid was used to recover the dye that had adhered to the biofilm. The absorbance at 630 nm was then used to examine the recovered solution. (**a**) Negative control; (**b**) 0.3 g/L CZEO; (**c**) 0.4 g/L CZEO; (**d**) 0.5 g/L CZEO; (**e**) 0.6 g/L CZEO; and (**f**) positive control (2 mg/L vancomycin). The inhibition rates of biofilms were presented as means ± standard deviations. Significance tests were based on the values obtained relative to the negative control and were considered statistically significant at * *p* < 0.05.

MRSA employs an effective survival strategy by forming a protective biofilm at the site of infection, which increases antibiotic resistance and enhances tolerance to environmental stress. In this study, we evaluated the inhibitory effects of CZEO on MRSA biofilm formation using crystal violet staining, which is a standard method for quantifying biofilms. The experiment designed to inhibit MRSA biofilms applied the same CZEO concentration range (0.3 to 0.6 g/L) used in the previous studies examining floating cell proliferation and analyzing metabolites. Figure 2 illustrates the concentration-dependent inhibitory effects of CZEO on MRSA biofilms after 24 h of incubation. In the control group, a high absorbance at 630 nm was observed, indicating extensive biofilm formation. In the group treated with CZEO, concentration-dependent inhibition of biofilm formation was observed.

The 0.3 g/L CZEO treatment inhibited biofilm formation by 36.66% relative to the control group. In addition, the rate of inhibition increased in a concentration-dependent manner, with values of 56.50%, 77.38%, and 98.09% observed at 0.4 g/L, 0.5 g/L, and 0.6 g/L, respectively. The inhibition rate observed at 0.6 g/L indicated that this dose was sufficient to replace the positive control vancomycin (2 mg/L; inhibition rate of 98.07%) for biofilm inhibition.

**Figure 3:**
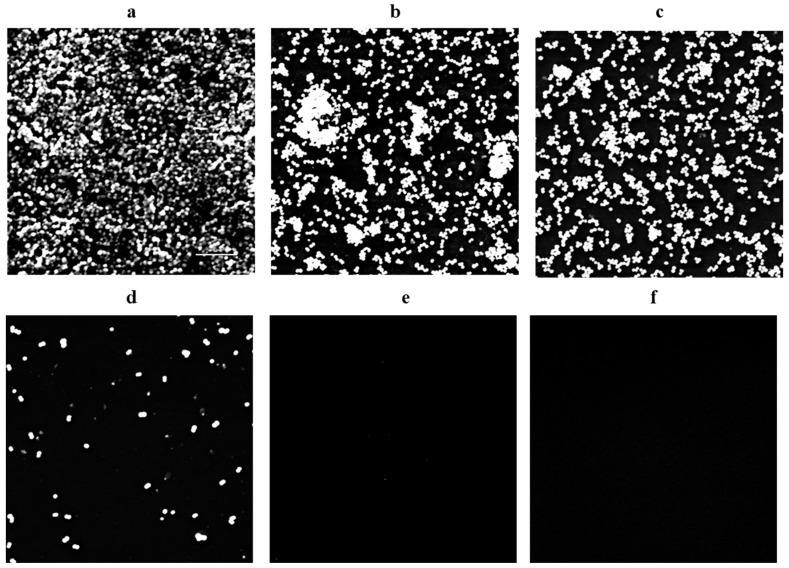
Analyzing the concentration-dependent MRSA biofilm removal effect of CZEO via scanning electron microscopy images. MRSA was inoculated in 1% glucose BHI medium that had been pretreated with CZEO and incubated for 24 h. To prevent structural modification of the biofilm, stabilization with a 2.5% glutaraldehyde solution was performed for 2 h. The samples were then subjected to stepwise dehydration with ethanol, and after complete drying overnight, the biofilm surface was platinum sputter coated to improve conductivity. Finally, the prepared samples were subjected to scanning electron microscopy to observe the microstructural changes in the MRSA biofilm. (**a**) Control; (**b**) 0.3 g/L; (**c**) 0.4 g/L; (**d**) 0.5 g/L; (**e**) 0.6 g/L; (**f**) positive control (2 mg/L vancomycin). Scale bar = 10 μm.

Figure 3 illustrates the impact of CZEO on the disruptive effect of MRSA biofilms, as observed through the use of scanning electron microscopy (SEM). In the control group, MRSA strains formed dense biofilms. In contrast, the CZEO-treated groups exhibited progressive disruption of the biofilm structure with increases in concentration. At a CZEO concentration of 0.3 g/L, biofilm damage was observed, while at 0.4 g/L, a further loss of biofilm structure and wider cell spacing were evident. At 0.5 g/L, most bacteria were no longer present, and only a few isolated cells remained. The 0.6 g/L CZEO treatment resulted in the near-total eradication of bacteria, indicating its potential as an alternative to the positive control vancomycin (2 mg/L).

### 2.4. CZEO Reduced the Survival Rate of MRSA

The bactericidal effect of CZEO against MRSA was evaluated using a LIVE/DEAD BacLight kit and confocal laser scanning microscopy (CLSM). The live cells were stained with SYTO™ (green), while propidium iodide (PI, red) was used to stain dead cells. In the planktonic MRSA assay, a bacteriostatic effect was observed at a concentration of 0.5 g/L of CZEO. To evaluate higher bactericidal efficacy, a concentration of 0.6 g/L was initiated, with a subsequent two-fold increase in concentration.

**Figure 4:**
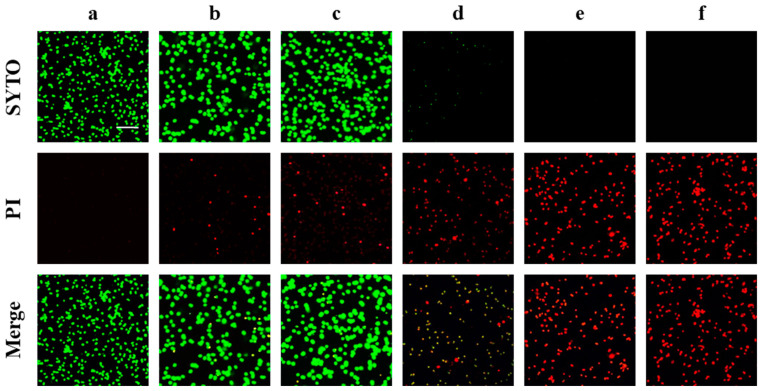
CZEO impairs the viability of MRSA. MRSA was treated at CZEO concentrations of 0.6, 1.2, 2.4, and 4.8 g/L for 30 min, followed by centrifugation and three washes with 0.85% saline to remove residual material. Subsequently, fluorescence staining was performed in the dark, and confocal laser scanning microscopy (CLSM) visualized the resulting material. (**a**) Control; (**b**) 0.6 g/L; (**c**) 1.2 g/L; (**d**) 2.4 g/L; (**e**) 4.8 g/L; and (**f**) positive control (2 mg/L vancomycin). Scale bar = 10 μm. PI, propidium iodide.

As illustrated in Figure 4, most cells in the control group exhibited green fluorescence. As the concentration of CZEO increased, the percentage of dead cells that exhibited red fluorescence significantly increased. In the 0.6 g/L CZEO treatment, red fluorescence increased slightly compared with the control, whereas in the 1.2 and 2.4 g/L CZEO treatments, the percentage of dead cells increased significantly. The CZEO treatment at a concentration of 4.8 g/L caused MRSA cell death, and live organisms were not visible. These findings supported the hypothesis that CZEO reduced MRSA viability in a concentration-dependent manner.

### 2.5. CZEO Downregulated All Pathogenic Genes in a Concentration-Dependent Manner

**Figure 5:**
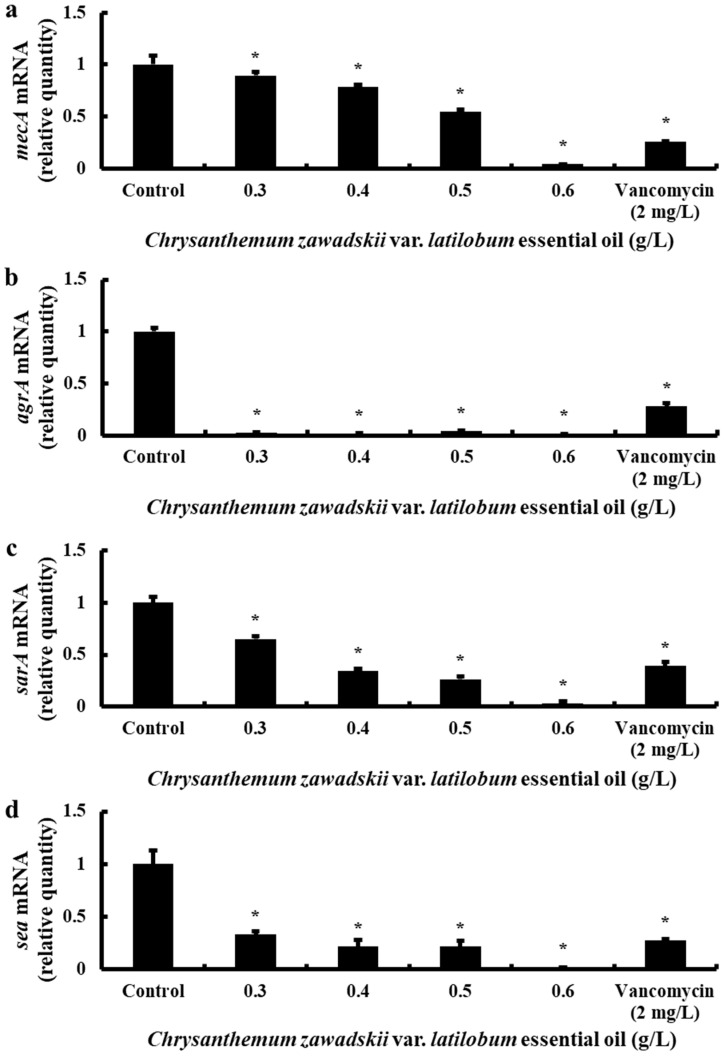
Inhibitory effect of CZEO on mRNA expression of MRSA virulence genes *mecA*, *agrA*, *sarA*, and *sea*. The efficacy of CZEO in treating MRSA was evaluated at concentrations of 0.3, 0.4, 0.5, and 0.6 g/L, and the mRNA expression levels of pathogenic genes were subsequently analyzed by real-time PCR. The genes analyzed included the following: (**a**) *mecA* mRNA expression level, (**b**) *agrA* mRNA expression level, (**c**) *sarA* mRNA expression level, and (**d**) *sea* mRNA expression level. The relative mRNA expression levels of virulence factors were measured and displayed as mean ± standard deviation. The level of significance was assessed at the * *p* < 0.05 level in comparison to the control, and statistically significant differences were observed.

We assessed the effect of CZEO on antibiotic resistance- and virulence-related gene expression in MRSA by examining the mRNA expression of *mecA*, *agrA*, *sarA*, and *sea* genes. CZEO was subjected to concentrations of 0.3, 0.4, 0.5, and 0.6 g/L, with vancomycin (2 mg/L) as a positive control. The data presented in Figure 5 illustrate the concentration-dependent inhibition of gene expression of all target genes.

The *mecA* gene was downregulated at CZEO concentrations exceeding 0.3 g/L and showed the most substantial downregulation at 0.6 g/L. The virulence regulatory genes *agrA* and *sarA* exhibited disparate responses, with strong inhibition observed at 0.3 g/L and 0.4 g/L CZEO, respectively. The toxin production-related *sea* gene showed a gradual decline in expression at CZEO concentrations exceeding 0.3 g/L and significant inhibitory effects at 0.6 g/L.

Thus, CZEO effectively inhibited the expression of genes related to antibiotic resistance (*mecA*), virulence factor regulation (*agrA* and *sarA*), and toxin production (*sea*) in MRSA.

## 3. Discussion

In a cross-sectional study of healthy adults, *S. aureus* was detected in a variety of body sites, including the nasal cavity and hands (27%), perineum (22%), forearms (20%), pharynx (10–20%), and axilla (8%) [20]. Although *S. aureus* is a normal colonizer of the human skin, nasal passages, and mucous membranes, it is recognized as a major pathogen that can cause skin diseases through toxin production [20,21,22,23]. *S. aureus* is an opportunistic pathogen that causes a number of infectious illnesses, despite its seemingly nonpathogenic appearance. Microorganisms can invade and cause localized soft-tissue infections during skin breakdown. Furthermore, bloodstream infections can result in severe complications, such as sepsis and endocarditis [24]. MRSA has demonstrated resistance to a range of β-lactam antibiotics, including methicillin and penicillin, leading to infections that are challenging to treat and have limited treatment options, including vancomycin [25]. This study aimed to investigate the antimicrobial activity of CZEO against MRSA and its effectiveness in inhibiting pathogenic factors.

The chemical composition of essential oils is dominated by isoprenoids (terpenes), although additional functional compounds are observed. Monoterpenes are the predominant constituents, while sesquiterpenes are the secondary constituents. Depending on the chemical functional group, these components are categorized into alcohols, aldehydes, epoxides, esters, ethers, ketones, and phenols. Although these compounds present differences in sensitivity between bacterial species, their antimicrobial activity is ordered phenols > aldehydes > ketones > alcohols > esters > hydrocarbons [26].

Essential oils have been used for more than 5000 years in various applications, including in cosmetics, food, insect repellents, and therapeutics. The chemical composition of essential oils can vary depending on the plant species and extraction technique [27].

The presence of double bonds, chirality, and oxygen atoms in monoterpenes contributes to the aroma of essential oils, and these molecules act as microbial defenses within the plant body [28]. Badawy et al. (2019) evaluated 25 monoterpenes against *E. coli* and antibiotic-resistant *S. aureus* and reported that the chemical species identified in the CZEO of this study presented antimicrobial activity. These species include camphor, camphene, α-pinene, linalool, limonene, α-terpineol, carvone, α-terpinyl acetate, myrcene, and thymol [29]. Sesquiterpenes represent a significant indicator of the Asteraceae family, to which *C. zawadskii* var. *latilobum* belongs, and are classified into caryophyllene, lactone, and eromophyllene sesquiterpenes based on their chemical structure and properties. Caryophyllene sesquiterpenes are characterized by a distinctive backbone consisting of a nine-membered ring and dimethylcyclobutene; in addition, β-caryophyllene and β-caryophyllene oxide exhibit antimicrobial and antifungal properties [30]. β-caryophyllene can compromise the cell membrane integrity of *Bacillus cereus*, inducing apoptosis, and impede biofilm formation and glucosyltransferase expression in *Streptococcus mutans* [31,32]. Furthermore, both β-caryophyllene and β-caryophyllene oxide can inhibit the growth of the plant pathogenic fungi *Sclerotinia sclerotiorum* and *Fusarium oxysporum* by up to 40% [33]. The chemical composition of CZEO is consistent with the findings of prior studies on essential oils, which reported a predominance of monoterpenes and a secondary predominance of sesquiterpenes. CZEO contains several compounds that have been reported to have antimicrobial activity in previous studies, suggesting that it may have antimicrobial effects against antibiotic-resistant bacteria.

Suspended MRSA exhibits higher pathogenicity and acute infection-associated factor expression than the biofilm form of MRSA [34]. The protein A, lipoteichoic acid, enterotoxin A, and other secretions stimulate fibroblasts to overproduce a variety of cytokines and growth factors, resulting in the increased secretion of interleukin-6, interleukin-8, vascular endothelial growth factor, transforming growth factor-β1, heparin-binding EGF-like growth factor, matrix metalloproteinase-1, matrix metalloproteinase-3, and others [34,35]. Such changes can interfere with the normal wound-healing process [34].

To investigate the inhibitory potential of concentration-specific CZEO treatments on the planktonic growth of MRSA, an assay was conducted to quantify the optical density (OD) of bacterial cultures prepared using a stepwise dilution method.

The experimental data revealed concentration-dependent growth inhibition, as shown in Figure 1. These findings indicate that CZEO effectively inhibits the growth of planktonic MRSA at concentrations exceeding 0.5 g/L. In vitro studies demonstrated that CZEO has the capacity to effectively inhibit the growth of suspended MRSA (IC50 = 0.39 g/L), suggesting its potential for the control of MRSA-associated infections.

During aerobic metabolism of MRSA, pyruvate, the end product of glycolysis, is converted to acetyl-CoA via a decarbonylation reaction [36]. Acetyl-CoA is then converted to acetyl phosphate, which is used to produce acetate and ATP. During the logarithmic growth phase of bacteria, excess acetate is expelled from the cells [36,37]. Acetate is involved in ATP production through the acetate kinase pathway, which is essential for energy production and biofilm formation. These metabolic pathways are essential for the survival of MRSA under various environmental conditions [37].

In this study, to evaluate the effect of CZEO on acid production by MRSA, we incubated MRSA treated with CZEO at various concentrations from 0.3 to 0.6 g/L and measured the change in pH before and after incubation.

The results of the experiment demonstrated a concentration-dependent increase in pH in CZEO-treated cells compared with that of the control (Table 3). These results suggest that the CZEO treatment inhibits acid production during MRSA growth, which may indicate its effects on bacterial metabolic activity.

Biofilm-forming bacteria exhibit significantly elevated resistance to antibiotics compared with that of the same strain in a suspended state [38]. MRSA biofilms are primarily composed of extracellular polymeric substances (EPSs), including polysaccharides, proteins, and eDNA. The polysaccharide poly-β(1-6)-N-acetylglucosamine enhances cell-to-cell binding and antibiotic resistance. Biofilm-associated proteins provide structural stability to biofilms, whereas fibronectin-binding proteins (FnBPs) and staphylococcal protein A promote cell–cell adhesion and immune evasion. In addition, eDNA enhances adhesion and cohesion [39].

A study conducted by Clarridge in 2013 investigated the potential correlation between MRSA nasal colonization and subsequent wound infections and revealed that 80% of 80 pairs of nasal and wound isolates were from the same strain, suggesting that MRSA colonization may increase the risk of infection [40]. Microbial biofilms are commonly found in chronic wounds, and MRSA within biofilms induces elevated levels of TNF-α secretion from fibroblasts compared with that of MRSA in suspension; thus, MRSA-based biofilms impair the wound healing process [34].

Biofilms consisting of EPSs enhance the antibiotic resistance and survival of MRSA within the host by strengthening intercellular cohesion and facilitating immune evasion. In this context, the present study aimed to determine the effects of CZEO on MRSA biofilm formation.

The concentration-dependent inhibitory effect of CZEO on biofilms formed on cell culture plates was evaluated using crystal violet staining. CZEO exhibited a concentration-dependent inhibitory effect on MRSA biofilms (Figure 2). At lower concentrations, complete inhibition is challenging due to the complex biofilm structure and occurrence of antibiotic resistance. However, at a high concentration (0.6 g/L), these defense mechanisms were overcome. This illustrates the potential value of CZEO as a natural product-based alternative for the management of MRSA colonization.

The formation of MRSA biofilms is a process that occurs in five distinct phases: attachment, colony formation, substrate production, maturation, and dispersal. In the initial phase, MRSA attaches to biotic (host) or abiotic surfaces via surface proteins, such as microbial surface components that recognize adhesive matrix molecules (MSCRAMMs). Subsequently, bacteria undergo progressive multiplication and produce extracellular polymeric material, including eDNA, to form microcolonies. During the maturation phase, bacterial cells release eDNA via autolysis to maintain substrate stability. Some cells enter a dormant state via a quorum-sensing system, thereby increasing antibiotic resistance. They also repress settlement genes in host tissues through AGR operons and activate proteins associated with tissue damage and autolysis. Finally, mature biofilms are prepared for the dispersal of some cells into a planktonic state by the expression of surfactant-like peptides and initiation of new biofilm colonies on other surfaces [41,42].

The scanning electron microscopy images presented in Figure 3 provide visual confirmation of the effect of CZEO on the structural integrity of MRSA biofilms. In the control group (Figure 3a), a dense biofilm structure and abundant extracellular matrix were identified, indicating that the initial attachment and maturation process of the biofilm was smooth and MRSA had a strong biofilm-forming ability. In contrast, concentration-dependent structural disruption of the biofilm was observed in the CZEO treatment group.

These findings suggest that CZEO may affect the integrity of MRSA biofilms. However, further mechanistic studies are required to determine whether CZEO has a direct effect on specific structural determinants of MRSA biofilms.

Fluorescence assays employing SYTO 9 and PI dyes are commonly used to evaluate bacterial viability. Most bacteria do not exhibit intrinsic fluorescence activity; therefore, specialized dyes are required to induce fluorescent signals. Such signals can be utilized to effectively characterize bacterial populations using instruments such as fluorescence microscopes, microplate readers, and flow cytometers [43].

The cell membrane state is a crucial indicator of the alive or dead status of bacteria. Intact cell membranes are selectively permeable, allowing the passage of certain substances while preventing the passage of others. In contrast, damaged cell membranes lose selective permeability. PI is a useful marker of dead cells because it can only enter cells with broken membranes and attach to DNA. In contrast, SYTO 9 can penetrate all cells and bind to nucleic acids, and it is excited at a blue wavelength of 485 nm and emits green fluorescence at 498 nm for DNA and at 501 nm for RNA. When both dyes are used simultaneously, PI exhibits a preferential binding to DNA over SYTO 9 [44,45].

As illustrated in Figure 4, the CLSM images demonstrated that CZEO effectively reduced the viability of MRSA cells. As PI is only able to permeate a cell if the cell membrane is damaged, the increase in PI-positive cells observed after CZEO treatment suggests that this treatment compromised the cell membrane integrity of MRSA. However, further studies are required to determine the exact molecular mechanisms by which CZEO induces cell membrane damage.

The primary cause of antibiotic resistance in MRSA is the expression of the *mecA* gene. PBP2a is encoded by this gene and located in a mobile genetic element known as the staphylococcal cassette chromosome mec. In addition, PBP2a has a lower affinity for β-lactam antibiotics [46,47] because it attaches to and blocks the activity of PBPs, which are involved in peptidoglycan formation in the bacterial cell wall. β-lactam antibiotics usually impair the construction of cell walls, which compromises bacterial structural integrity and ultimately results in cell death when peptidoglycan cross-linking of the cell wall is disrupted [46]. However, in MRSA, PBP2a, which is expressed by the *mecA* gene, circumvents antibiotic action [46,47]. PBP2a has an unusual structure that differs from that of other PBPs, thereby limiting its interaction with β-lactam antibiotics. The acylation reaction rate of PBP2a to β-lactam antibiotics is markedly reduced, which limits the formation of acyl-enzyme intermediates. These properties allow MRSA to persist in the synthesis of its cell wall, even when exposed to β-lactam antibiotics, thereby facilitating the acquisition of antibiotic resistance [47].

The *agrA* gene plays a pivotal role in MRSA pathogenicity control mechanisms because it is a principal component of the accessory gene regulator (*agr*) system [48,49]. Activation of the *agrA* gene is closely associated with an increased propensity for bacterial colonization. Once the threshold concentration of the autoinducing peptide is reached outside the cell, the *agrC* receptor recognizes it, thereby initiating the phosphorylation of *agrA*. *agrA* then stimulates the synthesis of RNAII and RNAIII by binding to the P2 and P3 promoter regions upon phosphorylation. The *agr* system components are generated by the *agrBDCA* operon, which is encoded by the RNA II transcript. This results in the formation of a self-amplifying regulatory circuit that accelerates system activation. RNA-III is a pivotal regulatory RNA that plays a crucial role in controlling the expression of multiple pathogenicity factors [48]. The *agrA* gene is regulated by RNAIII, which in turn controls the production of various toxins, including α-hemolysin and β-hemolysin. These toxins can destroy red blood cells, thereby increasing bacterial penetration and interfering with the host immune system, which in turn causes tissue damage. In particular, these toxins facilitate bacterial propagation at the infection site by destroying host cells and accelerating the spread of infection through tissue damage [49]. The *agrA* gene plays a role in the breakdown of mature biofilm structures and dispersal of bacteria. The *agrA* gene induces the production of phenol-soluble modulins (PSMs), which facilitate the breakdown of mature biofilms. Additionally, the *agrA* gene increases the production of nucleases and serine proteases, thereby accelerating the biofilm substrate degradation and cell dispersal [49].

*sarA* directly regulates the expression of MRSA pathogenicity factors and binds to the P2 promoter of the *agr* system to promote toxin production [48]. Kim et al. employed a DNA affinity capture assay to confirm the binding of *sarA* to the promoter regions of *mecA*, *sarA*, and *sarR* and showed that a mutant strain devoid of the *sarA* gene (Δ*sarA*) exhibited diminished resistance to oxacillin, a decreased capacity for biofilm formation, and a lower ability to produce pivotal pathogenicity factors, including PSM and α-hemolysin [50]. This diminished capacity for biofilm formation is linked to the overproduction of extracellular proteases. Typically, *sarA* suppresses the activity of these enzymes and stimulates the expression of FnBPs, thereby enhancing biofilm stability [51].

The *sea* gene has been identified as a considerable contributing factor to MRSA-based foodborne and immune-related illnesses. The gene is delivered by bacteriophages and activated in response to environmental stressors [52,53,54]. Staphylococcal enterotoxin A (SEA), encoded by the *sea* gene, is one of the most frequently reported enterotoxins. It remains stable under extreme environmental conditions, including high temperatures and proteolytic enzyme activity [52,53]. The pathogenicity of SEA occurs through two principal mechanisms. SEA is a single-chain protein structurally composed of two major domains: A and B. The A domain is a superantigen that binds with high affinity to MHC class II molecules on antigen-presenting cells. The aforementioned binding interaction engages T-cell receptors, thereby activating a multitude of T-cells and consequently disrupting the host immune system. This phenomenon culminates in the release of copious quantities of cytokines, which can potentially result in severe symptoms, such as toxic shock syndrome [52,54]. Additionally, SEA stimulates mast cells to secrete serotonin, which interacts with nerve cells to cause vomiting, one of the principal symptoms of food poisoning [54].

In this study, we evaluated the effects of CZEO on antibiotic resistance and pathogenicity-related gene expression in MRSA using quantitative PCR. The results demonstrated that CZEO significantly decreased the mRNA expression of *mecA*, *agrA*, *sarA*, and *sea* in a dose-dependent manner (Figure 5). The decreased expression of the *mecA* gene, which encodes PBP2a and has been demonstrated to confer β-lactam antibiotic resistance, suggests that CZEO may be involved in regulating antibiotic resistance in MRSA. In addition, the reduced expression of the *agrA* and *sarA* genes, which function as regulators in toxin production and biofilm formation, and the *sea* gene, which encodes enterotoxin A, suggests that CZEO may also help regulate MRSA’s pathogenicity. However, further studies are required to ascertain the precise molecular mechanisms by which CZEO inhibits the expression of these genes and identify changes at the protein level.

## 4. Materials and Methods

### 4.1. Extraction and Sample Preparation of CZEO

Fresh flowers (petals and pistils) of *C. zawadski* var. *latilobum* (Maxim.) Kitam were collected from mountainside grasslands in Wanggung-myeon, Iksan-si, Jeonbuk, Republic of Korea, at the full-flowering stage in late October 2022. The samples were authenticated by Professor Byung-Kil Choo (Department of Crop Agriculture and Life Science, Jeonbuk National University, Jeonbuk, Republic of Korea). A voucher specimen (CZL-20221028) was stored at the Laboratory of Fermentation Technology (Professor Myung-Kon Kim, Jeonbuk National University). Fresh samples were kept in an airtight plastic container and stored in a cold room (4 °C) for one day until use.

One kilogram of fresh flowers was mechanically ground and then subjected to water vapor distillation to extract the essential oil using a Clevenger-type distillation apparatus for 3 h. The essential oil yield was 0.17% of the wet weight of the raw material. A proportion of the extracted CZEO was allocated for GC–FID and GC–MS analyses, while the residual sample was stored for subsequent bacteriological testing.

To match the active concentration (0.3 to 4.8 g/L) of CZEO in the brain heart infusion (BHI; Difco Laboratories, Detroit, MI, USA) broth, 6 to 96 mg of CZEO was dissolved in 100 μL of dimethyl sulfoxide (DMSO; Sigma-Aldrich, St. Louis, MO, USA). The solution was then sonicated for 20 min and stored at −20 °C. Immediately before bacterial testing, 5 μL of the CZEO solution was added per 1 mL of BHI broth to achieve a DMSO concentration of 0.5% in the medium. An equivalent volume of DMSO was also added per 1 mL of BHI broth to generate the negative control for all bacterial tests, thus preventing DMSO from having any effects on the experimental design.

### 4.2. Analytical Techniques

#### 4.2.1. Gas Chromatography-Based Component Separation

A Hewlett-Packard 6890 series gas chromatograph (Hewlett-Packard, Palo Alto, CA, USA) was used to separate the sample components. The instrument was equipped with a FID and operated in 1:30 split-injection mode. Two distinct capillary columns were employed for analysis: non-polar DB-1 and polar DB-Wax. Both columns exhibited identical dimensions: 30 m length, 0.25 mm inner diameter, and 0.25 μm film thickness. The temperature control protocol was as follows: initial temperature of 40 °C that increased at a rate of 2 °C per min until reaching 230 °C, which was maintained for 20 min. The temperature of the injectors and detectors was maintained at 250 °C. Nitrogen was employed as the mobile phase, with a flow rate of 1 mL/min. Quantitative analysis of each component was conducted based on the peak areas obtained using an electronic integrator, resulting in the relative proportions of the individual components.

#### 4.2.2. Component Identification with Gas Chromatograph–Mass Spectrometer-Coupled Systems

For the structural analysis of the components, a system combining an Agilent Technologies 7890A gas chromatograph (Agilent Technologies, Santa Clara, CA, USA) with a 5975C mass-selectivity detector was used. The system operates in electron impact ionization (EI) mode, with an ionization energy of 70 eV. DB-1 and DB-Wax capillary columns were employed for the separation process, and they had the following dimensions: 30 m in length, 0.25 mm inner diameter, and 0.25 µm film thickness. Temperature control was identical to that used in the aforementioned gas chromatography analysis. The temperature of the sample inlet and ion source was set to 250 °C. Helium was used as the mobile-phase gas at a flow rate of 1 mL/min. The individual compounds were identified using two parallel methods (Table 4). Initially, the obtained mass spectra were compared with those in the NIST/NBS database [55], and the calculated retention indices were compared with those in the literature [56]. A straight-chain paraffin standard mixture of C_6_ to C_26_ was used as a reference for calculating the retention index [57].

### 4.3. In Vitro MRSA Culture and Inoculum Preparation

The in vitro optimized MRSA growth environment was adapted from the original methodology with minor modifications to align with the study objectives [58,59]. The distributed MRSA ATCC 33,591 (American Type Culture Collection, ATCC, Manassas, VA, USA) was streaked with a flame-sterilized platinum loop on 2.5 mg/L oxacillin-coated blood agar plates (KisanBio, Seoul, Korea) and incubated for one day at 37 °C under aerobic conditions. A suspension culture was then initiated, which consisted of three to five colonies in BHI broth containing the antifungal agent amphotericin B (2.5 mg/L; Life Technology Co., Grand Island, NY, USA) and the susceptible antibiotic oxacillin (2 mg/L; Sigma–Aldrich, Saint Louis, MO, USA). Culture was performed for 24 h under 37 °C with appropriate oxygen and humidity conditions. Before each experiment, the cultures were diluted to a concentration of 1 × 10^8^ colony-forming units (CFU)/mL using a spectrophotometer (Bio-Rad Laboratories Inc., Irvine, CA, USA).

### 4.4. Analysis of MRSA Multiplication and Acidity Generation

The procedures for measuring the proliferative activity and secreted acid of MRSA bacteria were slightly modified from the guidelines set forth by the Clinical and Laboratory Standards Institute for Broth Dilution Assays [60]. In 24-well plates, CZEO was diluted with BHI medium at concentrations of 0.3, 0.4, 0.5, and 0.6 g/L, and a 2 mg/L vancomycin treatment served as the positive control. MRSA was uniformly inoculated at a concentration of 5 × 10⁵ CFU/mL and incubated at 37 °C under aerobic conditions. Following a 24 h incubation period, the broth was suspended and dispensed in 200 μL aliquots into 96-well plates. To measure MRSA proliferation activity, the OD was measured at 550 nm using a microplate spectrophotometer. The extent of bacterial growth inhibition was quantified using the following equation:

(1) $$\mathrm{Bacterial}\;\mathrm{Growth}\;\mathrm{Inhibition}\;\mathrm{Rate}\;(\%)=\left(1-\frac{{OD}_{550\;nm}(CZEO-treated\;group)}{{OD}_{550\;nm}(Control\;group)}\right)\times 100$$

To quantify acid secretion by MRSA, MRSA was inoculated into BHI medium at a concentration of 5 × 10⁵ CFU/mL. The experimental groups were treated with varying concentrations of CZEO or vancomycin, whereas the negative control group remained untreated. The cultures were then incubated under aerobic conditions at 37 °C for 24 h. Following incubation, acid production in MRSA culture supernatants was evaluated using a calibrated pH sensor (Corning, Inc., Corning, NY, USA).

### 4.5. Assessment of MRSA Biofilm Formation Utilizing Crystal Violet Staining Methodology and Scanning Electron Microscopy

In this study, we adapted and modified the experimental methodology proposed by Ball et al. (2022) to analyze MRSA biofilms [61]. BHI medium with 1:100 glucose was treated with 0.3 to 0.6 g/L of CZEO and dispensed into 35 mm culture dishes (Corning, Inc., Corning, NY, USA) at a volume of 5 mL. The diluted MRSA cultures were uniformly added to each dish and incubated aerobically for one day (the inoculated cell density per dish was 5 × 10⁵ CFU/mL). The bacterial layer at the bottom of the dish was left intact; however, the medium was completely removed by suction, and the cells were rinsed gently twice with phosphate-buffered saline (PBS) at pH 7.4 (Gibco Laboratories, Grand Island, NY, USA). Subsequently, the samples were stained with 1% crystal violet (Becton, Dickinson and Company, Sparks, MD, USA) for one minute, and then residual dye was removed using PBS. The dishes were then dried on a sterile workbench (Jeio Tech, Seoul, Korea) and photographed for subsequent visualization. The reagents present within the MRSA biofilm were eluted with 30% acetic acid (Sigma-Aldrich, Saint Louis, MO, USA), and the wavelength (630 nm) was measured according to the thickness of the MRSA biofilm using a spectrophotometer (Bio-Rad Laboratories Inc., Irvine, CA, USA).

The degree of MRSA biofilm inhibition was quantified using the following equation:

(2) $$\mathrm{MRSA}\;\mathrm{Biofilm}\;\mathrm{Inhibition}\;\mathrm{Rate}\;(\%)=\left(1-\frac{{OD}_{630\;nm}(CZEO-treated\;group)}{{OD}_{630\;nm}(Control\;group)}\right)\times 100$$

The bacteria were cultivated under conditions identical to those described above for the MRSA biofilm assay. The biofilm layer formed on the bottom of the 35 mm dishes was fixed for 2 h by adding 5 mL of 2.5% glutaraldehyde solution (Sigma-Aldrich).

The lactam antibiotic-resistant bacterial layer was dehydrated with increasing ethanol concentrations at 10 min intervals, starting with 50% ethanol, and dried overnight in a desiccator cabinet (Sanplatec Corp., Osaka, Japan). Before performing SEM (JSM-6360, Jeol, Tokyo, Japan) observations, the surface of the dried biofilm was sputter-coated with a platinum thin film.

### 4.6. Bacterial Nucleic Acid-Binding Fluorescent Staining Assay in MRSA

The detection of live MRSA was evaluated using a LIVE/DEAD^®^ BacLight™ bacterial viability kit (Molecular Probes, Eugene, OR, USA). The test group was treated with CZEO at concentrations of 0.6, 1.2, 2.4, and 4.8 g/L in BHI medium and subsequently inoculated with the MRSA cultures (1 × 10^8^ CFU/mL). Similarly, the positive control was treated with vancomycin to facilitate comparisons of the test group’s behavior, whereas the negative control was inoculated with the broth alone, thereby exposing the bacteria to either drug. Polypropylene tubes (2 mL; Corning Inc.) were incubated at 37 °C in an aerobic environment with sufficient humidity for 30 min. Subsequently, after centrifugation at 6000 rpm and 4 °C for 7 min, the supernatant was removed and resuspended in 0.85% saline. Cells were collected under the same centrifugation conditions. This rinsing process was repeated twice, and 1 mL of 0.85% saline was dispensed to remove any residual influence of the medium, CZEO, or vancomycin. Then, 3 µL of fluorescent dye solution was added to each sample according to the manufacturer’s instructions. Cells were cultured in a dark room with a fluorescent dye. The stained specimens were pipetted into eight-microliter portions onto glass slides, and three-dimensional fluorescence images were collected using an LSM 510 confocal laser scanning microscope (Zeiss, Oberkochen, Germany).

### 4.7. Quantitative Assay of Pathogenic Factor Gene Expression in MRSA via Real-Time PCR

The concentration of CZEO was set to 0.3–0.6 g/L, which is within the range of antimicrobial effectiveness. BHI broth (10 mL) was inoculated with 1 × 10^9^ CFU/mL of MRSA and incubated aerobically at 37 °C for 24 h. Following the incubation period, the cells were harvested by centrifugation at 4 °C and 3000 rpm for 5 min, after which they were washed three times with sterile phosphate buffer. Following the manufacturer’s recommendations, total RNA was extracted using TRIzol^®^ reagent (Life Technologies, Carlsbad, CA, USA) and purified using the method of de Souza et al., which was optimized for this study [62]. Extracted RNA was purified by sequentially processing with chloroform, isopropanol, and 70% ethanol, with vortexing and centrifugation (12,000 rpm, 4 °C, 10 min) at each step. Purified RNA was dissolved in 20 μL of RNase-free water (USB Corporation, Cleveland, OH, USA) and then quantified at 260 nm using a BioSpec-nano spectrophotometer (Shimadzu Co., Kyoto, Japan), with its purity assessed using the A260/A280 ratio. In addition, the A260/A230 ratio was used to determine any organic contamination. A PrimeScript^TM^ RT Reagent Kit (Takara Bio, Kusatsu, Japan) was used to reverse transcribe the total RNA according to the manufacturer’s instructions. The synthesized cDNA was subjected to real-time PCR analysis using a StepOnePlus Real-Time PCR System (Applied Biosystems, Foster City, CA, USA) after preparing the mixture with Power SYBR^®^ Green PCR Master Mix (Applied Biosystems).

The DNA amplification reaction started with an initial denaturation step at 95 °C for 5 min, followed by 40 cycles of amplification. Each cycle consisted of a denaturation step at 95 °C for 15 s, an annealing step at 60 °C for 1 min, and an extension step at 72 °C for 30 s. The relative expression of MRSA virulence factor genes was analyzed using the 2^−ΔΔCt^ method, and the expression was normalized using *16s* rRNA as a housekeeping gene. The primer sequences are shown in Table 5.

### 4.8. Statistical Analysis

Non-parametric statistical analyses were conducted on the data collected from the experiments to evaluate the antimicrobial effects of CZEO on MRSA. All experimental results are shown as the mean values ± standard deviations. A Mann–Whitney U test was used to identify discrepancies between the control and experimental groups, while the Kruskal–Wallis test was used to assess the overall divergence between the various concentrations of essential oils.

Levene’s test was conducted to assess the homogeneity of variance among all groups. All statistical analyses were conducted using Microsoft^®^ Excel^®^ for Microsoft 365 MSO Version 2412 (Microsoft Corporation, Seattle, WA, USA), with *p* < 0.05 indicating significance.

## 5. Conclusions

This study focused on the chemical composition of CZEOs and identified 88 distinct compounds, which were classified into monoterpenes (68.82%; 38 compounds), sesquiterpenes (17.82%; 28 compounds), and others (5.01%; 22 compounds). CZEO demonstrated noteworthy antibacterial, antibiofilm, and bactericidal activities against MRSA, a multidrug-resistant pathogen. In particular, it significantly inhibited the expression of four virulence genes, *mecA*, *agrA*, *sarA*, and *sea*, which are involved in antibiotic resistance and pathogenicity. The *mecA* gene encodes PBP2α, which causes methicillin resistance; *agrA* and *sarA* are key regulatory genes that control virulence factors and biofilm formation, and *sea* encodes enterotoxin A, which causes food poisoning. The inhibition of these genes is crucial for reducing the infectivity and virulence of the pathogen, thereby effectively inhibiting the antibiotic resistance mechanism of MRSA and the production of virulence factors. Nonetheless, the findings of this study are confined to in vitro results, and further validation is required for clinical application. Consequently, although the efficacy of CZEO in inhibiting MRSA has been demonstrated, future long-term safety and toxicity studies are required.

## Figures and Tables

**Table 1:** Composition of the essential oil from the flowers of *Chrysanthemum zawadskii* var. *latilobum*.

Retention Indices		Compounds	Peak Area (%) ^c^
Apolar ^a^	Polar ^b^		
788	1087	*n*-Hexanal	0.11
849	1380	*cis*-Hexen-1-ol	0.14
862	1356	*n*-Hexanol	0.09
887	-	Santene	0.10
920	1009	Tricyclene	0.28
928	1029	α-Thujene	0.14
931	1027	α-Pinene	1.15
948	1070	Camphene	6.33
964	1123	Sabinene	0.54
973	1110	β-Pinene	0.44
979	-	1,2,4-Trimethylbenzene	0.06
985	1167	Myrcene	0.08
1008	1181	α-Terpinene	0.43
1016	1276	*ρ*-Cymene	0.47
1019	1196	Limonene	0.19
1021	1122	β-Phellandrene	0.19
1023	1213	1,8-Cineole	2.66
1073	1430	*ρ*-Cymenene	0.44
1086	1283	α-Terpinolene	0.18
1091	1427	α-Thujone	1.94
1095	1441	β-Thujone	0.40
1101	1537	Linalool	0.33
1123	1518	Camphor	39.69
1129	1721	*trans*-Sabinol	0.08
1130	-	*trans*-Verbenol	0.04
1136	1564	Pinocarvone	0.10
1149	1701	Borneol	2.23
1157	1763	*cis*-Chrysanthenol	2.79
1164	1662	*ρ*-Mentha-1,5-dien-8-ol	0.22
1168	1599	Terpinen-4-ol	0.07
1173	1697	α-Terpineol	0.18
1179	1798	Myrtenol	0.15
1191	1845	*cis*-Carveol	0.10
1200	1200	Dodecane	0.13
1207	1796	Cuminaldehyde	0.13
1211	1732	Carvone	0.14
1218	-	*cis*-*3*-Hexeny lisovalerate	0.24
1221	1725	Piperitone	0.29
1246	1569	*cis*-Chrysanthenyl acetate	0.50
1267	1575	Bornyl acetate	5.42
1274	-	*trans, cis*-2,4-Decadienal	0.12
1280	2212	Thymol	0.07
1288	-	Myrtenyl acetate	0.07
1301	-	*trans*-Carvyl acetate	0.15
1326	1679	α-Terpinyl acetate	0.11
1354	2164	Eugenol	0.09
1368	1493	α-Copaene	0.65
1373	1526	Berkheyaradulen	0.04
1383	1582-	β-Elemene	0.33
1392	-	α-Gurjunene	0.07
1400	1400	Tetradecane	0.04
1410	1590	*trans*-β-Caryophyllene	0.85
1449	1646	*cis*-β-Farnesene	0.27
1469	1737	Germacrene D	0.42
1479	1783	*ar*-Curcumene	0.25
1483	1720	β-Selinene	0.31
1492	1714	Zingiberene	0.09
1667	1456	*trans*-β-Farnesene	0.16
1500	1500	Pentadcecane	0.11
1510	1752	δ-Cadinene	0.15
1516	1775	β-Sesquiphellandrene	0.33
1563	2020	*trans*-Caryophyllene oxide	0.26
1566	1943	1,5-Epoxysalvial-4(14)-ene	1.22
1569	-	Globulol	0.38
1571	2118	Spathulenol	1.30
1577	-	Salvial-4(14)-en-1-one	0.12
1586	2043	Viridiflorol	0.22
1589	-	Guaiol	0.29
1600	1600	Hexadecane	0.59
1605	2088	Cubenol	0.59
1609	2167	Torreyol	1.44
1614	2218	β-Eudesmol	0.93
1620	2180	τ-Muurolol	0.45
1630	2223	α-Cadinol	5.77
1645	2210	α-Bisabolol	0.28
1657	2244	*cis,cis*-Farnesol	0.24
1700	1700	Heptadecane	0.13
1712	2368	Chamazulene	0.10
1714	2330	α-Cyperone	0.41
1800	1800	Octadecane	0.38
1830	2128	6,10,14-Trimethyl-2-pentadecanone	0.47
1900	1900	Nonadecane	0.52
2000	2000	Eicosane	0.08
2100	2100	Heneicosane	0.32
2000	-	Docosane	0.04
2300	2300	Tricosane	0.66
2400	-	Tetracosane	0.05
2500	2500	Pentacosane	0.54
Total			91.65

^a^ Retention index on an apolar DB-1 column. ^b^ Retention index on a polar DB-Wax column. ^c^ Peak area percentage is on an apolar DB-1 column.

**Table 2:** Chemical classification of *Chrysanthemum zawadskii* var. *latilobum* (Maxim.) Kitam Essential Oil.

Category	Sub-Class	Compound	Total (%)
Monoterpenes	Hydrocarbons	Santene, Tricyclene, α-Thujene, α-Pinene, Camphene, Sabinene, β-Pinene, Myrcene, α-Terpinene, *ρ*-Cymene, Limonene, β-Phellandrene, *ρ*-Cymenene, α-Terpinolene	10.96
	Alcohols	Linalool, *trans*-Sabinol, *trans*-Verbenol, Borneol, *cis*-Chrysanthenol, *ρ*-Mentha-1,5-dien-8-ol, Terpinen-4-ol, α-Terpineol, Myrtenol, *cis*-Carveol, 1,8-Cineole, Thymol	8.92
	Aldehydes	Cuminaldehyde	0.13
	Esters	*cis*-Chrysanthenyl acetate, Bornyl acetate, Myrtenyl acetate, *trans*-Carvyl acetate, α-Terpinyl acetate	6.25
	Ketones	α-Thujone, β-Thujone, Camphor, Pinocarvone, Carvone, Piperitone	42.56
Sesquiterpenes	Hydrocarbons	β-Elemene, α-Gurjunene, *trans*-β-Caryophyllene, *cis*-β-Farnesene, Germacrene D, *ar*-Curcumene, β-Selinene, Zingiberene, *trans*-β-Farnesene, β-Sesquiphellandrene, α-Copaene, Berkheyaradulen, δ-Cadinene	3.92
	Alcohols	Globulol, Spathulenol, Viridiflorol, Guaiol, Cubenol, Torreyol, β-Eudesmol, τ-Muurolol, α-Cadinol, α-Bisabolol, *cis*, *cis*-Farnesol	11.89
	Ketones & epoxides	Salvial-4(14)-en-1-one, α-Cyperone, *trans*-Caryophyllene oxide, 1,5-Epoxysalvial-4(14)-ene	2.01
Others	Alkanes	Dodecane, Tetradecane, Pentadcecane, Hexadecane, Heptadecane, Octadecane, Nonadecane, Eicosane, Heneicosane, Docosane, Tricosane, Tetracosane, Pentacosane	3.59
	Alcohols and aldehyde	*n*-Hexanal, *cis*-Hexen-1-ol, *n*-Hexanol, *trans*, *cis*-2,4-Decadienal	0.46
	Esters	*cis*-3-Hexenyl isovalerate,	0.24
	Miscellaneous	1,2,4-Trimethylbenzene, Chamazulene, 6,10,14-Trimethyl-2-pentadecanone, Eugenol	0.72

**Table 3:** *Chrysanthemum zawadskii* var. *latilobum* essential oil (CZEO) and its ability to suppress the acidogenicity of methicillin-resistant *Staphylococcus aureus* (MRSA).

Concentration (g/L)	^a^ Pre-Incubation pH (Mean ± SD)	Post-Incubation pH (Mean ± SD)	^b^ ΔpH (Acid Production)	^c^ Inhibition Rate (%)
Control	7.46 ± 0.00	5.96 ± 0.01	1.50	0%
0.3	7.45 ± 0.00	6.53 ± 0.02 *	0.92	38.58%
0.4	7.46 ± 0.01	6.80 ± 0.04 *	0.65	56.54%
0.5	7.44 ± 0.01	7.13 ± 0.03 *	0.31	79.38%
0.6	7.44 ± 0.01	7.40 ± 0.01 *	0.05	96.90%
Vancomycin (2 mg/L)	7.46 ± 0.00	7.41 ± 0.02 *	0.05	96.45%

The pH values are presented as the mean ± standard deviation based on the experimental results. A statistically significant difference was observed between the experimental group and control group after cultivation (* *p* < 0.05). ^a^ Pre-incubation is defined as the stage preceding both the incubation period and inoculation of microorganisms. ^b^ ΔpH was calculated as the difference between the initial pH (before cultivation) and final pH (after 24 h of cultivation) for each treatment group. ^c^ The inhibition rate was calculated using the following formula: Inhibition rate (%) = [(ΔpH control − ΔpH treatment)/ΔpH control] × 100, where ΔpH control is the pH change in the control group and ΔpH treatment is the pH change in the CZEO or vancomycin-treated groups.

**Table 4:** Instrumental Conditions for GC and GC–MS Analysis.

Parameter	GC Analysis Conditions				GC–MS Analysis Conditions				
Instrument	Hewlett-Packard 6890 Series GC				Agilent Technologies 7890A GC, 5975C Mass Selective Detector				
Detector	Flame Ionization Detector (FID)				Mass Selective Detector (EI mode, 70 eV)				
Column (Dimensions)	DB-1, DB-wax (30 m × 0.25 mm i.d., 0.25 μm film thickness)				DB-1, DB-wax (30 m × 0.25 mm i.d., 0.25 μm film thickness)				
Column Temperature	Unit	°C/min	Temp. (°C)	Hold (min)	Unit	°C/min	Temp. (°C)	Hold (min)	
	Initial	-	40	-	Initial	-	40	-	
	Ramp 1	2	230	20	Ramp 1	2	230	20	
Injector Temperature	250 °C				250 °C				
Detector Temperature	250 °C				Not applicable				
Ion Source Temperature	Not applicable				250 °C				
Carrier Gas	Nitrogen, 1 mL/min				Helium, 1 mL/min				
Component Identification	Comparison with the retention time (RT) position and literature				Comparison with NIST/NBS mass spectral database and literature				

**Table 5:** Primers for real-time PCR detection of pathogenic genes in methicillin-resistant *Staphylococcus aureus* (MRSA).

Gene	Sequences (5′→3′)		Tm (°C)	Base Count (nt)	GC Ratio (%)
*agrA*	Forward Primer	TGATAATCCTTATGAGGTGCTT	53.7	22	36.36
	Reverse Primer	TGATAATCCTTATGAGGTGCTT	55.6	21	42.86
*mecA*	Forward Primer	GTTAGATTGGGATCATAGCGTCATT	58.1	25	40
	Reverse Primer	TGCCTACTCATGTGTTCCTGTAT	59	27	37.04
*sarA*	Forward Primer	TGTTATCAATGGTCACTTATGCTG	56.3	24	37.5
	Reverse Primer	TCTTTGTTTTCGCTGATGTATGTC	57.1	24	37.5
*sea*	Forward Primer	ATGGTGCTTATTAGGTGTATC	50.1	21	33.33
	Reverse Primer	CGTTTCCAAAGGTAGTGTTATT	52.9	21	38.1
*16s rRNA*	Forward Primer	ACTGGGATAACTTCGGGAAA	55.2	20	45
	Reverse Primer	CGTTGCCTTGGTAAGCC	54.9	17	58.82

## Data Availability

The original contributions presented in this study are included in the article. Further inquiries can be directed to the corresponding authors.
